# Construction and application of a professional ethics assessment framework for university teachers in China

**DOI:** 10.3389/fpsyg.2026.1811590

**Published:** 2026-08-11

**Authors:** Fei Liu, Shuiping Zhu

**Affiliations:** Nantong University, Nantong, China

**Keywords:** assessment framework, professional ethics, scale development, teacher ethics development, university teachers

## Abstract

**Introduction:**

Clarifying the conceptual boundaries and assessment dimensions of university teachers’ professional ethics is a prerequisite for strengthening ethical standards in higher education. This study aimed to examine the connotation and constituent elements of university teachers’ professional ethics, develop a systematic assessment framework, and preliminarily evaluate its applicability.

**Methods:**

An integrated qualitative and quantitative research design was employed. On the basis of theoretical analysis and empirical investigation, a two-dimensional assessment framework was developed by combining psychological components with relational domains. The resulting framework was subsequently applied in a survey of 1,230 university teachers, and teacher self-evaluations were compared with student evaluations. Differences in professional ethics across teacher groups were also examined.

**Results:**

The assessment framework comprised four psychological components—moral cognition, moral emotion, moral volition, and moral behavior—and four relational domains: teacher–student relations, teacher–institutional collective relations, teacher–teaching relations, and teacher–academic research relations. These dimensions generated a total of 50 specific assessment items. The survey results indicated that the participating university teachers generally reported relatively high levels of professional ethics. Nevertheless, discrepancies emerged between teacher self-evaluations and student evaluations, and significant variation was observed across different teacher groups.

**Discussion:**

The proposed framework provides a multidimensional approach to conceptualizing and assessing the professional ethics of university teachers by integrating psychological processes with major professional relationships. The preliminary findings also highlight the importance of incorporating multiple evaluative perspectives and attending to group differences. Based on the framework development process and the assessment results, corresponding strategies are proposed to promote the professional ethics of university teachers.

## 1 Introduction

As the Chinese saying goes, “When students feel close to their teacher, they come to trust the way the teacher teaches.” Rooted in China’s long-standing historical and cultural tradition of respecting teachers and valuing education, teachers have been regarded as important moral exemplars in society (Ye and Law, 2019). University teachers, in particular, shoulder the responsibility of cultivating high-level modern talent for the nation and fulfilling the fundamental educational mission of fostering virtue. However, in recent years, incidents of moral misconduct allegedly involving some university teachers—such as exploiting students, engaging in academic misconduct, making inappropriate remarks, or maintaining improper relationships with students—have occasionally appeared in the media (Parnther, 2020). These incidents have exposed university teachers’ morality to “trial by public opinion” on social media and, in some cases, even to stigmatization.

Academic research on university teachers’ professional ethics has largely focused on basic pathways, the current state of development, countermeasures and recommendations for strengthening teacher ethics and professional conduct in higher education, as well as the causes of and reflections on teachers’ moral misconduct. However, such studies are mostly conducted in the form of experiential summaries and theoretical reflection, lacking in-depth theoretical analysis of the connotation of teacher ethics in higher education. Moreover, their conclusions often lack empirically grounded qualitative analysis. Although some studies have examined teachers’ professional moral conduct in China and recent work has begun to develop teacher ethics assessment tools in specific subject areas (Feng, 2007; Liu et al., 2022), research on university teachers’ professional ethics still lacks a systematic assessment framework and psychometrically validated measurement tools. In view of this, the present study constructs an evaluation model of professional ethics for university teachers on the basis of theoretical deduction, qualitative analysis, and practical exploration, and, guided by relevant psychometric theory, develops the Professional ethics Scale for University Teachers, with the aim of truthfully and comprehensively revealing the current status of professional ethics among university teachers in China and providing useful insights for strengthening teacher ethics development in Chinese higher education institutions (DeVellis and Thorpe, 2021).

## 2 Exploring the structure of university teachers’ professional ethics

A prerequisite for strengthening teacher ethics in higher education institutions is to clarify “what constitutes the ethics of university teachers,” that is, to specify the concrete connotation of university teachers’ ethics. This emphasis is consistent with higher education ethics research, which argues that ethical teaching requires conceptual clarification of professional responsibility, integrity, and the normative expectations embedded in academic practice (Macfarlane, 2004; Maxwell and Schwimmer, 2016). The present study constructs a measurement model of university teachers’ professional ethics at both the theoretical and operational levels. The theoretical level mainly concerns the conceptual connotation of teacher ethics in higher education, whereas the operational level primarily concerns the conceptual extension of teacher ethics in higher education.

### 2.1 Construction of the theoretical model

Generally speaking, university teachers’ ethics can be further distinguished into two major aspects: teacher ethics in a broad sense and teacher ethics in a narrow sense. The former refers to teachers’ political stance, social responsibility, and related dimensions, which fall under general civic morality; the latter specifically refers to teachers’ professional cultivation and belongs to professional ethics at a more specific level. In the Chinese context, teacher morality is often shaped by both institutional expectations and teachers’ professional self-understanding, reflecting a tension between regulation and autonomy (Ye and Law, 2019). The present study mainly discusses teacher ethics in the narrow sense. In higher education, professional ethics is closely related to teachers’ roles, responsibilities, duties, rights, and institutional obligations within the university context (Sethy, 2018). A review of the literature shows that existing studies often define university teachers’ professional ethics as “the basic norms and codes of conduct for university teachers,” yet this definition only reflects the practical function of teacher ethics and does not reveal its specific connotation (Campbell, 2000). Other studies, from the perspective of constituent elements, define it as “the sum of moral qualities, behavioral norms, and ideological concepts formed by university teachers in specific educational practice” (Campbell, 2003). Compared with the former, the latter further identifies three major components of university teachers’ professional ethics— “qualities,” “behaviors,” and “ideological concepts”—involving not only the explicit level of “moral behavior,” but also implicit elements such as “qualities” and “ideological concepts.”

Based on existing research and in combination with the classic psychological framework of the four-part division of “cognition, emotion, will, and behavior,” the present study defines university teachers’ professional ethics as the sum of moral cognition, moral emotion, moral volition, and moral behavior formed by university teachers in teaching practice (Narvaez and Lapsley, 2009). Specifically, moral cognition and moral emotion correspond to the “ideological concepts” in the above definition: moral cognition refers to university teachers’ understanding of policy regulations and the behavioral requirements imposed on them by professional ethics; moral emotion refers to university teachers’ emotional experiences when others or their own behaviors comply with or violate policy regulations and professional ethics. Moral volition corresponds to the ‘moral qualities’ in the above definition, referring to the degree of determination with which university teachers uphold ethical bottom lines when confronted with moral decisions. Moral behavior represents the outward manifestation of the “behavioral norms” in the above definition, referring to university teachers’ practice of professional ethics and the extent to which their teaching and educating behaviors conform to moral norms (Campbell, 2003). Therefore, at the level of conceptualization, the present study constructs a theoretical model of teacher ethics in higher education that includes four ontological elements—moral cognition, moral emotion, moral volition, and moral behavior—among which moral cognition, moral emotion, and moral volition are the implicit components of university teachers’ ethics and are externally manifested through moral behavior.

### 2.2 Construction of the operational model

Although the above theoretical model further clarifies the ontological connotation of teacher ethics in higher education, it remains relatively abstract and lacks sufficient operability. It cannot directly guide or evaluate teachers’ professional conduct; therefore, it is necessary to further identify the specific domains in which teacher ethics in higher education is manifested. Because university teachers’ professional ethics is primarily reflected in their interactions with others and in the professional relations in which they are embedded, prior work on teachers’ professional ethics and codes of professional conduct suggests that a comprehensive conceptualization of university teachers’ professional ethics should involve four relational domains: the teacher–student relational domain, the teacher–institutional collective relational domain, the teacher–teaching relational domain, and the teacher–academic research relational domain (American Association of University Professors, 2009). Among these, the teacher–student relational domain includes teachers’ care for students’ lives and academic development, representing the primary manifestation of teacher love (*ShiAi*). The teacher–institutional collective relational domain includes teachers’ compliance with institutional regulations and their safeguarding of collective interests, representing the primary manifestation of teacher professionalism (*ShiYe*). The teacher–teaching relational domain includes teachers’ attitudes toward teaching and their continuous updating of teaching skills, representing the primary manifestation of teaching competence (*ShiNeng*). The teacher–academic research relational domain includes teachers’ compliance with research norms and academic ethics. This domain is relatively distinctive to university teachers compared with primary and secondary school teachers and represents the primary manifestation of scholarly practice (*ShiShu*).

**Figure 1:**
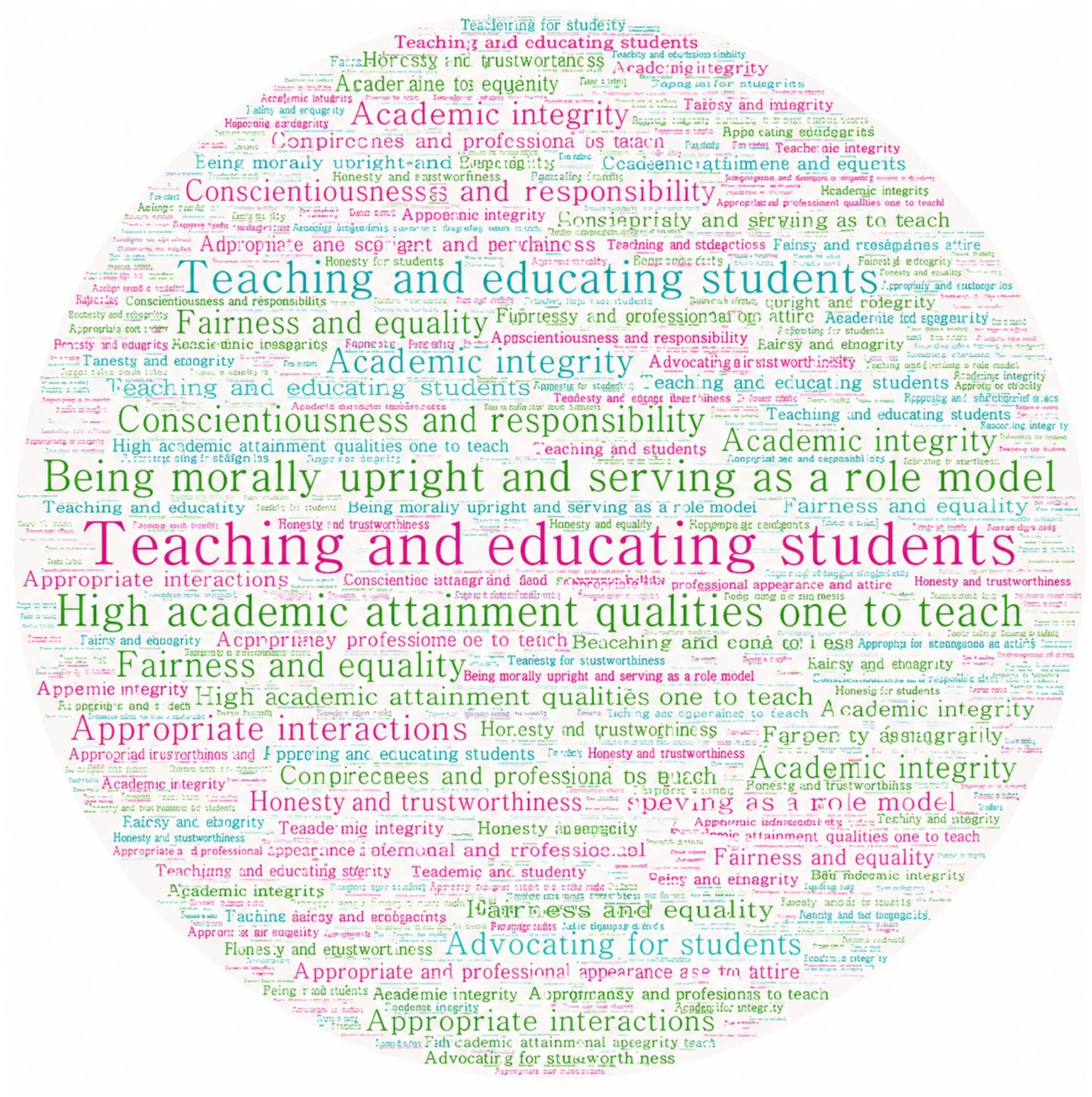
Manifestations consistent with teacher ethics.

**Figure 2:**
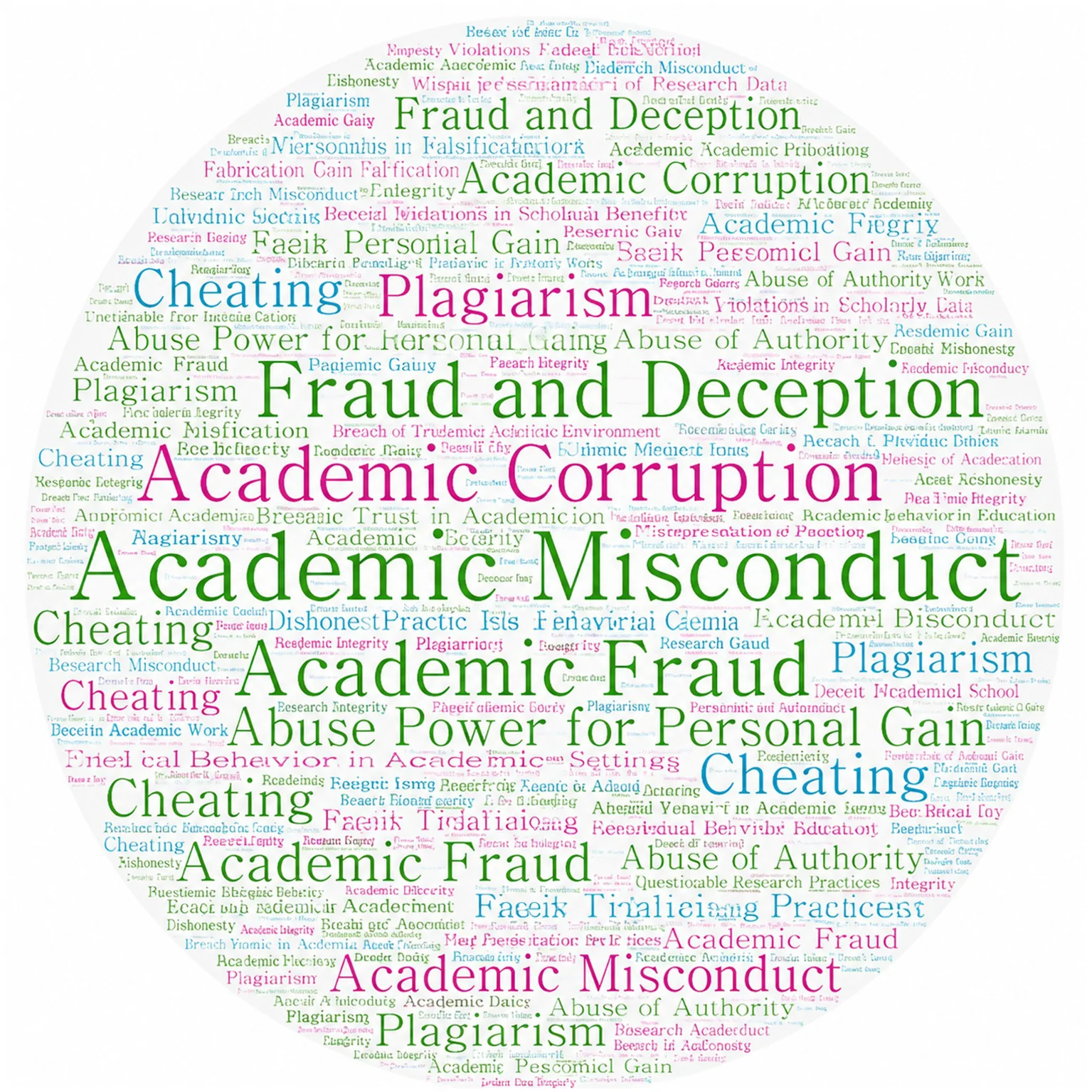
Manifestations that violate teacher ethics.

Semi-structured interviews were conducted with 230 university teachers and 274 college students to examine whether the proposed categorical division was reflected in participants’ accounts and to collect behavioral exemplars. The interview questions included: “In your view, in what aspects is university teachers’ professional ethics mainly manifested?” and “In your view, in what aspects are behaviors that violate university teachers’ professional ethics mainly manifested?” (Barrett et al., 2012). As shown in Table 1, Figures 1, 2, the collected materials were imported into NVivo 11.0 software for three-level coding. The qualitative results were generally consistent with the above literature analysis, providing preliminary support for conceptualizing university teachers’ professional ethics as being manifested across four relational domains: the teacher–student relational domain, the teacher–institutional collective relational domain, the teacher–teaching relational domain, and the teacher–academic research relational domain. Based on the collected behavioral exemplars, these four domains were, respectively, labeled as “caring for students,” “institutional responsibility,” “education and teaching,” and “academic research.”

**Table 1:** Results of grounded coding for university teachers’ professional ethics.

Selective coding	Axial coding (No. of Node References)	Open coding	Example reference content
Teaching	Teaching and educating students (316)	rigorous scholarship; imparting knowledge and resolving doubts; teaching according to students’ aptitude; student-centered approach; fostering virtue through education	Carefully preparing and delivering lessons; helping students with learning and career development; not only cultivating students’ professional knowledge and competencies, but also caring about their mental health
	Being morally upright and serving as a role model (92)	serving as a moral exemplar; teaching by words and deeds; noble moral character	Demonstrating exemplary conduct in words and actions; exerting influence on students subtly and imperceptibly
	High academic attainment qualifies one to teach (41)	broad learning and versatility; lifelong learning; professional competence	Deep mastery of disciplinary knowledge; keeping abreast of the latest developments in the research field
Students	Fairness and equality (174)	treating students equally; respecting students	Treating students equally; not insulting students; respecting students’ opinions
	Conscientiousness and responsibility (268)	caring for students; understanding students; helping students; patience and enthusiasm	Proactively caring for students; providing appropriate guidance; actively supporting students’ growth and success
	Appropriate interactions (53)	observing proper boundaries	Engaging in improper relationships with students; indecent assault; sexual harassment
Academic Research	Academic integrity (98)	seeking truth from facts	Plagiarism; tampering with or misappropriating others’ academic achievements; abusing academic resources and academic influence
	Pursuit of scholarship (68)	research competence; pursuit of truth; dedication to scholarship; academic rigor	Using one’s lifelong learning to pursue truth and devote oneself to research
Institutional Responsibility	Commitment and dedication to work (43)	abiding by laws and regulations; fulfilling duties conscientiously	Understanding and complying with institutional regulations; patriotism and law-abiding conduct; dedication to one’s post
	Justice and integrity (101)	not abusing power; not seeking personal gain	Using public office for private gain; unauthorized use of the university’s name, patents, venues, or other resources for personal benefit
	Unity and collegiality (36)	harmonious relations; healthy competition; cooperation	Education is a form of labor that requires strong collective coordination; teachers’ work involves competition, but even more importantly, cooperation

**Figure 3:**
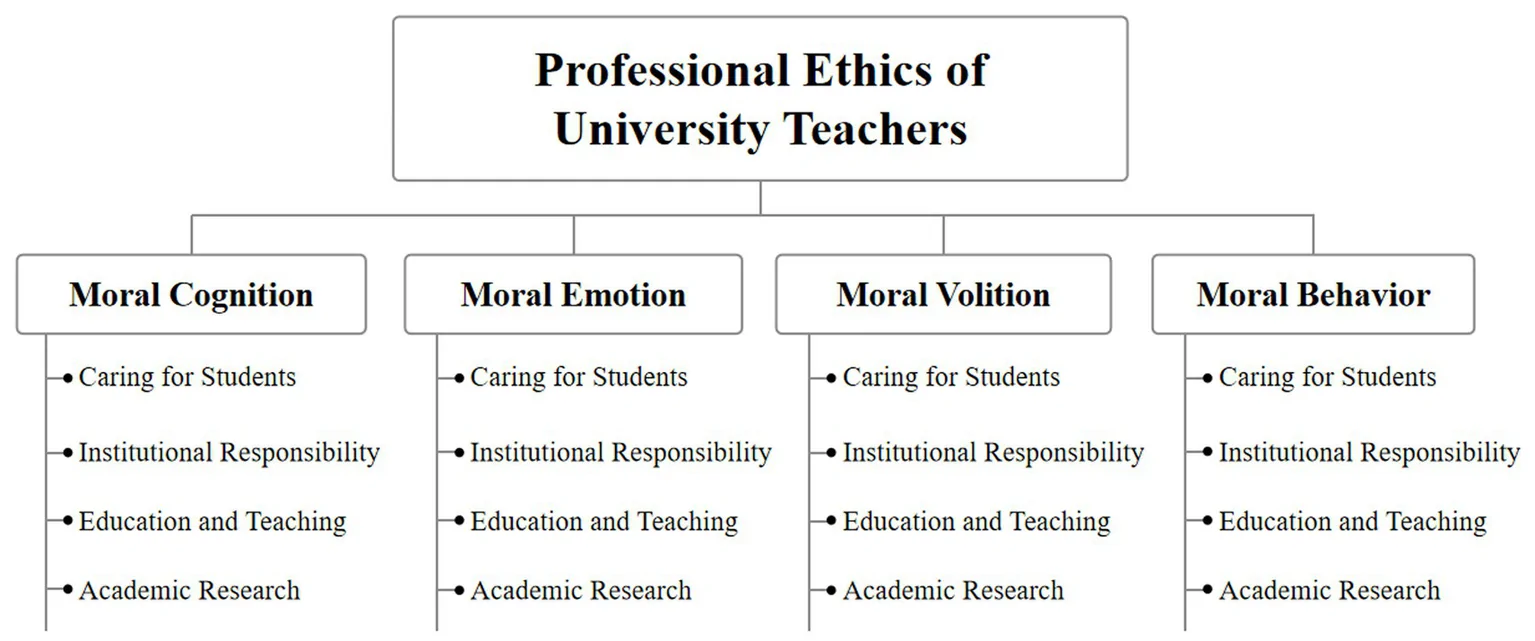
Two-dimensional framework of University Teachers’ Professional Ethics: Psychological Components × Relational Domains.

In summary, based on theoretical review, literature analysis, and qualitative coding, the present study constructs a two-dimensional measurement framework of university teachers’ professional ethics, integrating four psychological components with four relational domains. Specifically, moral cognition, moral emotion, moral volition, and moral behavior constitute four psychological components of university teachers’ professional ethics. Each psychological component is manifested across four relational domains: caring for students, institutional responsibility, education and teaching, and academic research. Thus, the proposed framework should be understood as a two-dimensional structure of “psychological components × relational domains,” rather than as a conventional first-order–second-order hierarchical model. The two-dimensional structure of the framework is shown in Figure 3.

## 3 Preliminary development of the professional ethics scale for university teachers

### 3.1 Principles for scale development

*Giving equal weight to ideals and bottom lines*: In the early stage, requirements for teacher ethics mainly advocated moral ideals, such as teachers’ admiration for the “spirit of the red candle.” With the occasional occurrence of moral misconduct among university teachers, relevant policy documents began to establish “red-line safeguards” for teacher ethics in higher education. For instance, the *Ten Guidelines for Professional Conduct for University Teachers in the New Era* clearly stipulates the bottom-line requirements for university teachers’ professional conduct. Therefore, the present study upholds the principle of “giving equal weight to ideals and bottom lines,” advocating teachers’ pursuit of noble morality (e.g., when students encounter difficulties in life or academic study, teachers should provide help without seeking return), while also setting “red-line safeguards” for their behavior (e.g., requesting money or property from students under various pretexts, or accepting gifts from students, constitutes behavior that violates professional ethical norms for teachers). It should be noted that ideal teacher ethics and bottom-line teacher ethics are not strictly oppositional, but complementary: the bottom line is the foundation, and the ideal represents sublimation.

*Synergy between self-discipline and external regulation*: As mentioned above, early requirements for teacher ethics primarily advocated moral ideals, requiring teachers to regulate their own professional ethics, which reflected a high degree of voluntariness and self-discipline. As cases of unethical conduct have gradually been exposed, policy documents have begun to impose explicit requirements on teacher ethics, shifting teacher ethics from self-discipline toward external regulation. Accordingly, the present study follows the principle of “synergy between self-discipline and external regulation,” emphasizing both university teachers’ self-restraint in professional conduct (e.g., I always pay attention to my appearance, speech, and behavior, setting a good example for students) and the regulation of their professional conduct through relevant provisions (e.g., In experiments or research, I am able to strictly follow established scientific procedures).

*Integration of theory and operability*: Here, “theory” refers to the psychological components of teacher ethics in higher education, namely moral cognition, moral emotion, moral volition, and moral behavior, whereas “operability” refers to the relational domains in which teacher ethics is manifested, namely the teacher–student, teacher–institutional collective, teacher–teaching, and teacher–academic research relational domains. Therefore, in constructing the model, the present study takes into account both the theoretical concept of teacher ethics in higher education and its operational level, ensuring that each specific assessment item is grounded in scientific theory while also being aligned with the real context of university teachers, thereby achieving both theoretical rigor and practical operability.

### 3.2 Preliminary item screening

Based on the behavioral exemplars collected from semi-open interviews and the literature analysis, and in light of the current occupational realities of university teachers in China, specific assessment items were developed (Boateng et al., 2018). After all items had been drafted, several experts in moral education, psychology, and education were invited to conduct critical revisions of each item. After repeated rounds of revision, the *Preliminary Professional ethics Scale for University Teachers* comprising 128 items was ultimately formed. The preliminary scale was developed according to the two-dimensional framework of psychological components × relational domains. It included four psychological-component subscales—moral cognition, moral emotion, moral volition, and moral behavior—and, within each subscale, items were designed to cover four relational domains: caring for students, institutional responsibility, education and teaching, and academic research. The preliminary scale adopts a 6-point scoring method, with scores from 1 to 6 representing “completely inconsistent” to “completely consistent,” respectively, and reverse-scored items are included.

## 4 Testing and establishment of the professional ethics scale for university teachers

### 4.1 Participants

In the first survey, the developed *Preliminary Professional ethics Scale for University Teachers* was used as the instrument to conduct an online assessment of 654 teachers from multiple universities in two provinces in mainland China, for exploratory factor analysis. A total of 632 valid questionnaires were collected, yielding an effective response rate of 96.64%. Among the participants, 305 were male teachers (48.3%) and 327 were female teachers (51.7%); 203 had a teaching tenure of 10 years or less (32.1%), 218 had a tenure of 10–20 years (34.5%), and 211 had a tenure of 20 years or more (33.4%); in terms of professional title, 260 were lecturers or teaching assistants (41.1%), 278 were associate professors (44.0%), and 94 were professors (14.9%).

In the second survey, the developed *Professional ethics Scale for University Teachers* was used as the instrument to conduct an online assessment of 1,266 teachers from multiple universities in two provinces in mainland China. A total of 1,230 valid questionnaires were collected, yielding an effective response rate of 97.16%. Among them, 566 were male teachers (46.0%) and 664 were female teachers (54.0%); 88 were teaching assistants (7.2%), 515 were lecturers (41.9%), 449 were associate professors (36.5%), and 178 were professors (14.5%); 432 had a teaching tenure of less than 10 years (35.2%), 438 had a tenure of 10–20 years (35.6%), and 360 had a tenure of more than 20 years (29.2%). A random subsample of 500 questionnaires was selected for testing the scale’s reliability and validity.

To reduce potential social desirability bias, all questionnaire surveys were administered anonymously. Participants were informed that their responses would be used only for academic research purposes and that there were no right or wrong answers. They were also encouraged to answer honestly according to their actual perceptions and experiences. These procedures were intended to reduce evaluative pressure and to encourage more truthful responses when reporting on professional ethics-related issues.

### 4.2 Exploratory factor analysis

#### 4.2.1 Item analysis

Item analysis was conducted using the 632 datasets from the first administration. First, based on the total scores, the scores of the four initial subscales were ranked from high to low. The top 27% and bottom 27% of cases were selected to form the high-score group and low-score group, respectively. Independent-samples *t* tests were performed on the mean score differences between the two groups for each item to examine item discrimination. The results showed that the mean scores of the two groups on all 128 items reached significant differences (*p* < 0.001). Second, item–total correlation analysis was conducted by calculating the correlation between each item and the total score of its corresponding subscale. Two items with correlations lower than 0.20 (C2 and C10) were deleted.

#### 4.2.2 Factor extraction

Consistent with the proposed two-dimensional framework, exploratory factor analysis was conducted separately for the four psychological-component subscales. The KMO measure and Bartlett’s test of sphericity indicated that the KMO values were 0.85 for moral cognition, 0.94 for moral emotion, 0.94 for moral volition, and 0.90 for moral behavior. The χ^2^ values of Bartlett’s test were 6812.59 (*p* < 0.001), 12191.89 (*p* < 0.001), 9454.11 (*p* < 0.001), and 8565.88 (*p* < 0.001), respectively, all reaching ideal levels. These results suggest that common factors exist among the items within each subscale, indicating suitability for further factor analysis. Principal component analysis with varimax rotation was employed to extract factors with eigenvalues greater than 1 (Worthington and Whittaker, 2006). Items that did not meet the following criteria were removed: (1) communality less than 0.3; (2) factor loading less than 0.5; (3) cross-loading (loadings greater than 0.4 on two or more factors, with a loading difference less than 0.2); (4) factors containing fewer than three items; and (5) items with obvious misclassification.

After multiple rounds of exploration and the deletion of items or factors that did not meet the criteria, four relational-domain factors with eigenvalues greater than 1 were extracted within each of the four psychological-component subscales of university teachers’ professional ethics. Specifically, 16 items were retained for the moral cognition subscale, 14 for the moral emotion subscale, 13 for the moral volition subscale, and 14 for the moral behavior subscale. These factors explained 60.54%, 68.72%, 64.79%, and 59.83% of the total variance of the corresponding subscales, respectively. Item loadings, variance contributions, and communalities are shown in Tables 2–5.

**Table 2:** Factor loadings of items in the moral cognition subscale (*n* = 632).

Item	Factor loading				
	1	2	3	4	Communality
A20	0.79				0.73
A18	0.77				0.61
A21	0.76				0.69
A17	0.65				0.46
A19	0.61				0.55
A29		0.83			0.76
A27		0.80			0.72
A25		0.65			0.44
A31		0.58			0.40
A28		0.56			0.47
A13			0.83		0.77
A14			0.82		0.75
A12			0.58		0.37
A2				0.83	0.73
A1				0.82	0.70
A3				0.64	0.55
Eigenvalue	4.93	1.97	1.54	1.24	Total
% of Variance Explained	30.83	12.33	9.64	7.74	60.54

**Table 3:** Factor loadings of items in the moral emotion subscale (*n* = 632).

Item	Factor loading				
	1	2	3	4	Communality
B21	0.87				0.81
B22	0.82				0.73
B19	0.81				0.73
B20	0.79				0.72
B17	0.65				0.50
B18	0.58				0.53
B28		0.77			0.71
B25		0.74			0.61
B30		0.69			0.57
B2			0.87		0.80
B1			0.86		0.81
B3			0.55		0.39
B10				0.90	0.87
B9				0.84	0.85
Eigenvalue	5.75	1.57	1.21	1.09	Total
% of Variance Explained	41.04	11.24	8.65	7.78	68.72

**Table 4:** Factor loadings of items in the moral volition subscale (*n* = 632).

Item	Factor loading				
	1	2	3	4	Communality
C7	0.80				0.67
C6	0.78				0.67
C1	0.76				0.60
C3	0.73				0.61
C14		0.86			0.75
C13		0.79			0.71
C15		0.72			0.68
C19			0.83		0.74
C18			0.82		0.67
C24			0.63		0.45
C27				0.78	0.74
C26				0.78	0.75
C31				0.60	0.37
Eigenvalue	4	2.21	1.13	1.08	Total
% of Variance Explained	30.79	17.09	8.71	8.27	64.79

**Table 5:** Factor loadings of items in the moral behavior subscale (*n* = 632).

Item	Factor loading				
	1	2	3	4	Communality
D20	0.87				0.79
D21	0.76				0.66
D19	0.74				0.58
D24	0.62				0.47
D29		0.78			0.61
D30		0.76			0.61
D31		0.66			0.53
D27		0.54			0.29
D4			0.84		0.76
D3			0.82		0.71
D6			0.58		0.47
D14				0.82	0.74
D13				0.78	0.62
D15				0.68	0.54
Eigenvalue	3.86	1.76	1.63	1.13	Total
% of Variance Explained	27.58	12.54	11.65	8.07	59.83

#### 4.2.3 Determination and attribution of factors

Within each of the four psychological-component subscales of the Professional Ethics Scale for University Teachers—moral cognition, moral emotion, moral volition, and moral behavior—four relational-domain factors with eigenvalues greater than 1 were extracted. These factors corresponded to four relational domains: the teacher–student relational domain, the teacher–institutional collective relational domain, the teacher–teaching relational domain, and the teacher–academic research relational domain. Based on the items contained in each factor, the four relational-domain factors were labeled as “caring for students,” “institutional responsibility,” “education and teaching,” and “academic research,” respectively. The factors and items under each subscale are shown in Table 6.

**Table 6:** Factor naming and number of items for each subscale.

Subscale	Relational-Domain Factor	No. of Items	Subscale	Relational-Domain Factor	No. of Items
Moral cognition	Caring for Students	3	Moral volition	Caring for Students	4
	Institutional Responsibility	3		Institutional Responsibility	3
	Education and Teaching	5		Education and Teaching	3
	Academic Research	5		Academic Research	3
Moral emotion	Caring for Students	3	Moral behavior	Caring for Students	3
	Institutional Responsibility	2		Institutional Responsibility	3
	Education and Teaching	6		Education and Teaching	4
	Academic Research	3		Academic Research	4

### 4.3 Reliability and validity testing

A random sample of 500 questionnaires was drawn from the 1,230 datasets in the second survey. Mplus 8.0 was used to conduct confirmatory factor analysis (CFA) sequentially for each subscale, and SPSS 24.0 was used for reliability testing.

CFA was conducted separately for the four subscales. Based on the modification indices (MI) from the CFA, it was found that four items in the moral cognition subscale (Items 3, 7, 8, and 14) and three items in the moral emotion subscale (Items 3, 6, and 7) showed relatively high collinearity with other items and were therefore removed. The fit indices of the revised models are shown in Table 7. The results indicate that the models for each subscale demonstrated good fit, suggesting that the scale has a sound structure and good construct validity.

**Table 7:** Results of confirmatory factor analysis for the subscales.

Scale	*χ^2^/df*	RMSEA	CFI	TLI	SRMR
Moral cognition subscale	3.88	0.08	0.96	0.95	0.05
Moral emotion subscale	3.82	0.08	0.98	0.97	0.03
Moral volition subscale	2.52	0.06	0.98	0.98	0.03
Moral behavior subscale	3.15	0.07	0.97	0.96	0.04

On the basis of the confirmatory factor analysis, the correlations among moral cognition, moral emotion, moral volition, and moral behavior were examined. The results showed moderate to high correlations among these psychological components (correlation coefficients ranging from 0.40 to 0.78), suggesting that they are related components of the broader construct of university teachers’ professional ethics. Therefore, taking the scores of moral cognition, moral emotion, moral volition, and moral behavior as observed variables, an exploratory factor analysis was conducted again. The results indicated that only one eigenvalue greater than 1 was extracted, explaining 66.74% of the total variance. This suggests that moral cognition, moral emotion, moral volition, and moral behavior can be understood as related psychological components of the broader construct of university teachers’ professional ethics.

Reliability analysis was conducted for the revised four subscales and their subordinate factors. The results showed that the internal consistency Cronbach’s *α* coefficient for the total *Professional ethics Scale for University Teachers* was 0.93. The Cronbach’s α coefficients for the four subscales were 0.80, 0.94, 0.83, and 0.86, respectively. The Cronbach’s α coefficients for the subordinate factors within each subscale ranged from 0.70 to 0.95, reaching a satisfactory level. These results indicate that the four subscales exhibit high reliability.

## 5 Application of the professional ethics scale for university teachers

### 5.1 Participants

The valid data from the 1,230 teachers in the second survey were used to analyze the current status of teacher ethics among university teachers in China. The participants had a mean age of 41.60 years (*SD* = 7.97), and detailed demographic information is provided in Section 4.1. In addition, 273 student questionnaires were collected and compared with teacher questionnaires for a comparative analysis.

### 5.2 Instruments

*Teacher questionnaire:* The revised *Professional ethics Scale for University Teachers* was used. It comprises four psychological-component subscales—moral cognition, moral emotion, moral volition, and moral behavior—with a total of 50 items. A 6-point Likert scoring method was adopted (1 = “completely inconsistent,” 6 = “completely consistent”). Higher scores indicate a higher level of teacher ethics.

*Student questionnaire:* The student questionnaire consisted of two items: “Overall, how would you evaluate the current level of professional ethics among university teachers?” and “Excluding the influence of social news media, rumors, and the like, based on your own experiences interacting with university teachers, how would you evaluate the level of professional ethics among university teachers?” A 5-point scoring method was used (1 = “very poor,” 5 = “very good”). To compare results with the teacher questionnaire, the scores were converted to a 6-point scale. It should be noted that these two items were designed to provide a descriptive comparison between students’ overall impressions and their evaluations based on direct personal experience, rather than to test the causal influence of social media or related news reports.

### 5.3 Data analysis

SPSS 21.0 was used to conduct descriptive statistical analyses and difference tests.

### 5.4 Results

#### 5.4.1 Descriptive analysis of university teachers’ professional ethics

The descriptive results in Table 8 show that the surveyed university teachers rated their own level of teacher ethics relatively high, with the total score and the mean scores of all dimensions exceeding 5. As shown in the descriptive results in Table 9, students’ mean rating based on their own direct experiences interacting with university teachers was 4.78, whereas their mean overall rating of the current level of university teachers’ professional ethics was 4.62. A paired-samples *t* test showed that the difference between the two ratings was significant, with experience-based evaluations being higher than overall evaluations.

**Table 8:** Descriptive statistics of university teachers’ self-evaluations of teacher ethics.

Variable	Min	Max	*M*	*SD*
Total teacher ethics score	2.16	6.00	5.57	0.49
Moral cognition	1.00	6.00	5.71	0.62
Moral emotion	1.00	6.00	5.80	0.55
Moral volition	3.23	6.00	5.37	0.74
Moral behavior	1.79	6.00	5.44	0.61

**Table 9:** Descriptive statistics and difference test of students’ evaluations of university teachers’ ethics.

Variable	*M*	*SD*	*t*	*p*
Overall evaluation	4.62	0.86	5.12	<0.001
Experience-based evaluation	4.78	0.93		

#### 5.4.2 Differences in university teachers’ professional ethics across demographic variables

(1) Gender differences in university teachers’ professional ethics.

As shown in Table 10, an independent-samples *t* test revealed significant gender differences in university teachers’ professional ethics, with female teachers scoring significantly higher than male teachers.

(2) Differences in university teachers’ professional ethics by professional title.

**Table 10:** Test of gender differences in university teachers’ professional ethics.

Variable	Category	*M*	*SD*	*t*	*p*
Gender	Male	5.53	0.51	−2.63	0.009
	Female	5.60	0.47		

As shown in Table 11, one-way analysis of variance (ANOVA) indicated that there were no significant differences in university teachers’ professional ethics across professional titles.

(3) Differences in university teachers’ professional ethics by teaching tenure.

**Table 11:** Test of differences in university teachers’ professional ethics by professional title.

Variable	Category	*M*	*SD*	*F*	*p*
Professional title	Teaching Assistant	5.52	0.55	0.61	0.606
	Lecturer	5.57	0.51		
	Associate Professor	5.58	0.44		
	Professor	5.55	0.49		

As shown in Table 12, one-way analysis of variance (ANOVA) revealed significant differences in university teachers’ professional ethics across teaching tenure groups. Specifically, teachers with 10–20 years of teaching experience scored significantly higher in professional ethics than those with less than 10 years of experience and those with more than 20 years of experience.

**Table 12:** Test of differences in university teachers’ professional ethics across teaching tenure groups.

Variable	Category	*M*	*SD*	*F*	*p*	*Post hoc* comparisons
Teaching tenure	≤10 years	5.54	0.54	4.99	0.007	1 < 2 3 < 2
	10–20 years	5.63	0.45			
	≥20 years	5.53	0.48			

## 6 Results and discussion

Based on theoretical review, literature analysis, and qualitative coding, the present study constructed a two-dimensional measurement framework of university teachers’ professional ethics, integrating four psychological components with four relational domains, and conducted a preliminary application of this framework.

The qualitative data provided preliminary support for identifying four psychological components of university teachers’ professional ethics: moral cognition, moral emotion, moral volition, and moral behavior, corresponding to the classic psychological framework of cognition, emotion, volition, and behavior. Among them, *moral cognition*, *moral emotion*, and *moral volition* are implicit components of university teachers’ professional ethics. They constitute deep-seated factors that generate professional ethical behavior among university teachers, and are also externally manifested through moral behavior. The four relational domains include the teacher–student relational domain, the teacher–institutional collective relational domain, the teacher–teaching relational domain, and the teacher–academic research relational domain, which together represent the major professional contexts in which university teachers’ professional ethics is manifested. The qualitative data provided preliminary support for this classification and further suggested that teachers and students tended to place particular emphasis on the teacher–student relational domain and the teacher–teaching relational domain when discussing university teachers’ professional ethics.

The quantitative analysis showed that, overall, the self-rated scores of the surveyed university teachers on professional ethics and all subscales were relatively high, all exceeding 5 points on a 1–6 scale, suggesting that the surveyed teachers generally reported a relatively high level of professional ethics. More specifically, scores for moral cognition and moral emotion were higher than those for moral volition and moral behavior. This suggests that university teachers in China have developed a clear understanding of the requirements of professional ethics, are able to make moral judgments regarding their own and other teachers’ professional conduct, and generate corresponding emotional responses; however, there remains room for improvement in the implementation of moral behavior, suggesting that there remains room for strengthening moral volition and translating ethical understanding into ethical conduct.

From a comparative perspective, there were substantial differences between students’ evaluations and teachers’ self-evaluations, indicating divergences between the student and teacher perspectives in assessing university teachers’ professional ethics. On the one hand, this may be because students and teachers do not attend to the same aspects of professional ethics: students may be more concerned with teaching practices and teacher–student interactions, whereas teachers’ self-evaluations may involve broader professional domains, such as institutional responsibility, academic research, and other aspects of professional conduct. On the other hand, professional ethics carries strong implications of social moral evaluation; thus, both teacher self-reports and student evaluations may be influenced by public moral judgments and socially shared impressions. In the present study, students’ evaluations based on direct personal experience were more favorable than their overall evaluations. However, this difference should be interpreted descriptively rather than causally. Because the two items differed in wording, referent, and cognitive task, and because media exposure was neither experimentally manipulated nor directly measured, the observed difference cannot be taken as evidence that social media or related news reports reduced students’ evaluations. Instead, it may reflect multiple factors, including differences in evaluation targets, item wording, social stereotypes, public moral judgments, and item-order effects.

In terms of demographic differences, the present study found that female teachers scored significantly higher than male teachers in professional ethics. This gender difference should be interpreted cautiously. One possible explanation is that gendered socialization, role expectations, and differences in mentoring or student-support experiences may shape teachers’ self-perceptions and reported professional ethical practices. However, because the present study did not directly measure these potential mechanisms, the observed gender difference should be understood as descriptive rather than explanatory. Future research should examine the psychological, institutional, and professional factors that may account for gender-related differences in teachers’ professional ethics. The present study also found no significant differences in professional ethics across professional titles among the surveyed university teachers, suggesting that professional title was not significantly associated with self-reported professional ethics in this sample. Further analysis showed significant differences in professional ethics by teaching tenure. Specifically, teachers with 10–20 years of teaching experience scored significantly higher in professional ethics than those with less than 10 years of experience and those with more than 20 years of experience. The fact that teachers with 10–20 years of tenure scored significantly higher than those with less than 10 years suggests that the development of professional ethics among university teachers is a process of cultivation: teachers who are newly entering the profession come to grasp the requirements of professional ethics more accurately through teaching practice and professional training, and gradually use these requirements to regulate their own professional conduct, resulting in a gradual upward trend in professional ethics. Meanwhile, the finding that teachers with 10–20 years of tenure scored significantly higher than those with more than 20 years may be related to the possibility that, as teaching years increase, some teachers experience job burnout. Burnout, particularly emotional exhaustion and depersonalization, has been widely shown to undermine teachers’ professional functioning and engagement (Maslach et al., 2001; Skaalvik and Skaalvik, 2010).

Nevertheless, several limitations should be acknowledged. First, because professional ethics is a highly value-laden construct, participants’ responses may have been influenced by social desirability. Although this study attempted to reduce such bias through anonymous administration and by incorporating both teacher self-evaluations and student evaluations, it did not include a standardized social desirability measure. Therefore, the possibility that teachers overestimated their own professional ethics, or that students’ evaluations were shaped by broader social expectations and public moral judgments, cannot be fully ruled out. Future research should incorporate social desirability scales, multi-informant assessments, and behavioral or institutional indicators to more rigorously examine and control for this potential bias. Second, the representativeness of the sample should be interpreted with caution. Although the participants were recruited from multiple universities and covered teachers with different genders, professional titles, and teaching tenures, all teacher participants were drawn from universities in two provinces in mainland China. Therefore, the sample cannot be considered nationally representative of all university teachers in China. Regional differences, institutional characteristics, disciplinary backgrounds, and university types may influence teachers’ professional ethics and their assessment results. Future research should further validate the proposed assessment model and scale using larger and more diverse samples across different provinces, institutional levels, and disciplinary fields, ideally with probability-based sampling strategies (Putnick and Bornstein, 2016).

## 7 Educational implications

### 7.1 Grasp the bottom line of teacher ethics and advocate moral ideals

The bottom line of teacher ethics refers to the normative requirements for university teachers’ professional ethics stipulated in various policy documents, whereas moral ideals further refer to teachers’ spontaneous pursuit of the “spirit of the red candle” and the like. The bottom line primarily functions through moral external regulation, constituting the basic requirement that teachers’ conduct conform to relevant norms; moral ideals operate through moral self-discipline, representing elevated expectations built upon the bottom line. In the early stage, norms of teacher ethics mainly emphasized the ideal nature of teacher ethics, guiding teachers’ actual speech and behavior through moral exemplars; however, the practical effect was not satisfactory, because abstract ideal requirements obscured the actual trajectory of the development and practice of teacher ethics. The idealized moral image lacked strong binding force for teachers and provided insufficient practical guidance, resulting in the phenomenon of an “overflow of ideals” but a “scarcity of rules.” With the occurrence of misconduct incidents involving some teachers, requirements for teacher ethics in higher education have gradually shifted from an ideal-oriented mindset to a bottom-line mindset, beginning to establish “red-line safeguards” through specific policy documents and to impose strict qualitative norms on professional conduct so as to strengthen constraints. However, if only the bottom-line mindset is emphasized, teachers’ professional ethics may be confined merely to a basic level, thereby losing the driving force for further ethical development. Therefore, efforts to strengthen teacher ethics in higher education should uphold both “bottom-line teacher ethics” and “ideal teacher ethics”: the bottom line safeguards the ideals, while the ideals guide the development of the bottom line. Accordingly, in practical measures for teacher ethics development, teachers’ professional conduct should not only be strictly regulated through relevant documents, but corresponding moral role models should also be established to guide teachers to develop from bottom-line ethics toward ideal ethics, and to shift from external moral regulation to moral self-discipline.

### 7.2 Strengthen moral practice to achieve the integration of knowledge and action

University teachers’ understanding of professional norms constitutes a deep foundation for translating such understanding into behaviors that conform to professional ethical standards. The implicit components of university teachers’ professional ethics (moral cognition, moral emotion, and moral volition) also need to be externally manifested through moral behavior. According to the present survey, university teachers in China already have a clear understanding of whether certain behaviors conform to or violate professional ethical norms, which may be attributed to earlier cognitively oriented publicity and education on teacher ethics during the process of teacher ethics development; however, teachers’ own practice of professional ethical behavior remains insufficient, reflecting a state of “knowing but not acting” or “knowing but not yet acting.” This suggests that efforts to strengthen teacher ethics in higher education should not stop at cognitive publicity, allowing professional ethics to remain merely at the abstract cognitive level, but should instead ground it in concrete teaching practice. Specifically, first, through irregular supervision of teaching and research, deviations in teachers’ professional conduct can be identified and corrected in a timely manner, thereby shaping professional behavior that conforms to norms; second, professional ethics practice activities can be held regularly, promoting the transformation of implicit moral cognition into concrete moral behavior through firsthand moral practice; third, teachers’ professional ethics is not only reflected at the macro level of teaching and research, but also in the micro-level daily interactions between teachers and students and among colleagues (Campbell, 2003; Shapira-Lishchinsky, 2011). Teachers are often able to uphold professional ethics at the macro level (e.g., adhering to research ethics), yet may neglect minor incidents in daily teaching (e.g., refraining from bringing personal emotions into the classroom), which manifests as a lack of moral sensitivity and insufficient firmness of moral volition. Therefore, through the cultivation of moral sensitivity and willpower, moral cognition can be further transformed into moral behavior.

### 7.3 Cultivate teacher ethics throughout the entire career process and alleviate job burnout

From the distribution of teaching tenure, university teachers’ professional ethics exhibits an overall trend of first rising and then declining. The upward trend in the early stage of employment indicates that teachers’ professional ethics is not an innate endowment, but rather develops through a process of cultivation. Early professional training and teaching practice play a positive role in promoting the improvement of university teachers’ professional ethics (Maxwell and Schwimmer, 2016). At the same time, this highlights the importance of such measures for cultivating teacher ethics, suggesting that in the process of strengthening teacher ethics in higher education, universities should provide necessary ethics training for novice teachers to facilitate their role transition and encourage them to regulate themselves in accordance with ethical norms for university teachers. In addition, the declining trend in the later stage suggests that negative factors such as job burnout may pose threats to efforts in strengthening teacher ethics in higher education. This implies that teacher ethics development in universities should not only attend to the characteristics of different stages of teachers’ career development and emphasize the cultivation of teacher ethics throughout the entire process, but also recognize that such efforts should not be confined solely to moral improvement. Attention should also be given to the interaction between other psychological factors and morality, so as to construct an integrated psychological system that promotes the development of teacher ethics.

## Data Availability

The data supporting the findings of this study are available from the corresponding author upon reasonable request.
